# Prevalence and population genetics of the emerging honey bee pathogen DWV in Chinese apiculture

**DOI:** 10.1038/s41598-019-48618-y

**Published:** 2019-08-19

**Authors:** Qingyun Diao, Dahe Yang, Hongxia Zhao, Shuai Deng, Xinling Wang, Chunsheng Hou, Lena Wilfert

**Affiliations:** 1 Institute of Apicultural Research, Chinese Academy of Agricultural Sciences, Beijing, 100093 P.R. China; 2 Key Laboratory of Pollinating Insect Biology, Ministry of Agriculture, Beijing, 100093 P.R. China; 3 Guangdong Key Laboratory of Animal Conservation and Resource Utilization, Guangdong Public Laboratory of Wild Animal Conservation and Utilization, Guangdong Institute of Applied Biological Resources, Guangzhou, 510260 P.R. China; 4 Centre for Ecology and Conservation, University of Exeter, Penryn Campus, UK; 5 Institute of Evolutionary Ecology and Conservation Genomics, University of Ulm, 89069 Ulm, Germany

**Keywords:** Ecological epidemiology, Evolutionary ecology, Evolutionary genetics

## Abstract

Honey bees are agriculturally important, both as pollinators and by providing products such as honey. The sustainability of beekeeping is at risk through factors of global change such as habitat loss, as well as through the spread of infectious diseases. In China and other parts of Asia, beekeepers rely both on native *Apis cerana* and non-native *Apis mellifera*, putting bee populations at particular risk of disease emergence from multi-host pathogens. Indeed, two important honey bee parasites have emerged from East Asian honey bees, the mite *Varroa destructor* and the microsporidian *Nosema ceranae*. As *V. destructor* vectors viral bee diseases, we investigated whether another key bee pathogen, Deformed Wing Virus (DWV), may also have originated in East Asian honey bee populations. We use a large-scale survey of apiaries across China to investigate the prevalence and seasonality of DWV in managed *A. mellifera* and *A. cerana* colonies, showing that DWV-A prevalence was higher in *A. mellifera*, with a seasonal spike in prevalence in autumn and winter. Using phylogenetic and population genetic approaches, we show that while China and East Asian DWV isolates show comparatively high levels of genetic diversity, these bee populations are not a source for the current global DWV epidemic.

## Introduction

Honey bees play a vital role in agriculture and food security through the pollination of crops as well as the production of honey and other related goods such as royal jelly and honey bee-collected pollen. While the proportion of global agricultural production relying upon animal pollination has nearly doubled in the last 50 years^1^, apiculture increasingly fails to provide sufficient pollination services in Europe^2^ and farmers in parts of Southwestern China have reportedly had to rely on hand-pollination due to pollinator declines^3^, a practice which is not economically viable. China alone currently hosts more than 9 million bee colonies and 180,000 beekeepers^4^. Honey bee populations in China are undergoing rapid changes, with a strong drive to intensify apiculture and increase the number of managed honey bees^5^, and a concurrent reduction in managed and wild *A. cerana* in line with the increasing use of *A. mellifera*^6^. Beekeepers in America and Europe have experienced high colony mortality in recent years^7,8^, which threaten the sustainability of beekeeping operations.

While habitat loss and pesticide exposure certainly play a major role, declines in honey bees have been exacerbated by emerging diseases^9,10^. Several field studies have linked overwinter mortality of honey bee hives to increased titers of Deformed Wing Virus (DWV)^11–14^, which in turn are driven by the presence of the ectoparasitic mite *Varroa destructor* (hereafter referred to as ‘Varroa’)^15–17^. DWV is a globally distributed single-stranded RNA Picornavirus that can infect honey bees and other insects such as bumble bees^18,19^. DWV and other pathogens can be transmitted between species through direct and indirect contact, such as by foraging on contaminated flowers or through behavior such as robbing (reviewed in^19,20^).

Varroa, itself an emerging parasite that originated in the East Asian honey bee *A. cerana*, feeds on the honey bee’s fat body^21^. It can thus transmit virus particles directly into the bee’s body cavity^22^. Field and laboratory studies have shown that the presence of Varroa leads to an increase in prevalence and titre of DWV in honey bees^15–17^. Prior to the emergence of this vector, DWV was considered largely avirulent in honey bees^23^. The high DWV titers associated with Varroa infestations^15,17^ result in an increase in virulence: high titer DWV infections may result in the wing deformities giving this virus its name (reviewed in^18^). The virus also reduces survival in individual infected workers as well as increasing mortality in the colony’s overwinter workforce^24,25^, thereby reducing honey bee fitness. DWV is currently undergoing a global epidemic, driven by Varroa-infested European honey bee populations^26^. The combined spread of DWV and its emerging vector Varroa has been facilitated by the unregulated global movement of honey bees between countries and the resulting DWV pandemic is spreading into wild pollinators^24,26^, threatening insect pollination.

Globally, the European honey bee *Apis mellifera* is currently predominantly used in apiculture and pollination service. In Asia, and in particular in China, beekeepers use the indigenous honey bee *A. cerana* in addition to non-native *A. mellifera*. The Asian honey bee has coevolved with Varroa mites (*Varroa jacobsoni* and *V. destructor*) and possesses behavioural^27–30^ and physiological resistance mechanisms against these mites^31^, which may protect them from the current global DWV epidemic^26^. At the same time, East Asia is the epicentre of disease emergence for bees: both the Varroa mite and the gut parasite *Nosema ceranae* have jumped from the Asian honey bee *A. cerana* to the European honey bee *A. mellifera* in East Asia in the last century and have spread globally. This raises the question of whether East Asian honey bee populations may serve as a source or reservoir for other emerging bee pathogens such as DWV.

Here, we test the hypothesis that Chinese managed *A. cerana* populations are part of the phylogenetic source population for the current global Varroa-associated DWV epidemic, as suggested by this Asian bee population’s role in the emergence of *N. ceranae* and in particular DWV’s novel vector, the Varroa mite *V. destructor*. Given *A. cerana’s* relative resistance to *V. destructor*, we expect lower DWV prevalence than in *A. mellifera*. If Chinese managed honey bees serve as a source for the current global DWV epidemic, we expect to find higher ancestral genetic variation in this DWV population. To test these predictions, we use a large-scale multi-year survey of both European and Asian honey bees across China. Using phylogenetic and population genetic approaches, we show that while China and East Asian DWV isolates indeed show comparatively high levels of genetic diversity, these bee populations are not the ancestral source for the current global DWV epidemic.

## Results

### Prevalence

50 *A. cerana* hives from 24 apiaries and 117 *A. mellifera* hives from 61 apiaries were sampled across 22 Chinese provinces over a 2 year period between April 2015 and March 2017 (see Tables S1, S2 for colony- and apiary-level data). The estimated true colony-level prevalence (i.e. the percentage of DWV-positive colonies) of DWV-A in Chinese *A. mellifera* populations reaches 45.7% (95% Confidence Interval (CI) 35.6–56.2%), whereas the true colony-level prevalence of DWV-A for *A. cerana* is estimated only at 5.6% (0–18.3%); this estimation takes into account the uncertainty of PCR-based pathogen detection, conservatively assuming a PCR assay sensitivity and specificity of 95%^32^. DWV is a viral complex, consisting of at least 3 viral strains, the globally distributed DWV-A, as well as DWV-B (originally described as Varroa Destructor Virus-1 (VDV-1) in *V. destructor*^33^) and the rare DWV-C^34^. Sequence analysis confirmed that these samples belong to DWV-A. We additionally screened for DWV-B using specific primers for the *rdrp*-gene. We found DWV-B in two *A. mellifera* colonies out of a total of 117, resulting in an estimated true colony-level prevalence of 0–0.83%; we found no evidence for DWV-B presence in the 50 *A. cerana* colonies tested. A phylogenetic tree (Fig. S1) shows that the non-heterozygous Chinese DWV-B isolate SC-3 was highly similar to the known European DWV-B isolates.

**Figure 1:**
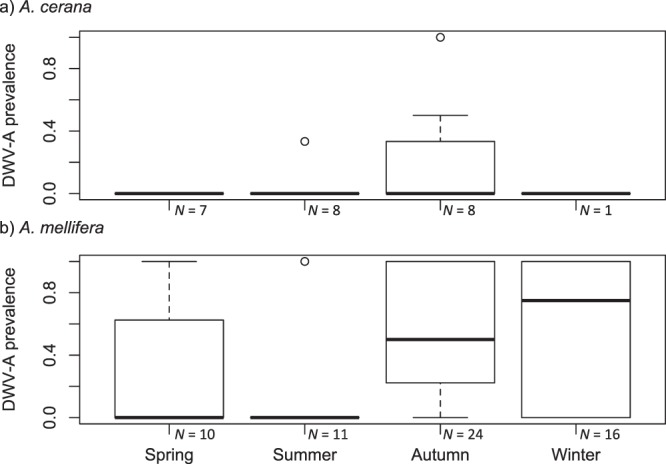
Apiary-level DWV-A prevalence in *A. cerana* and *A. mellifera* across seasons; Sample sizes indicate the number of apiaries per site per species (*N*_*apiaries*_ = 85, *N*_*hives*_ = 167). Both host species and season affect prevalence, with prevalence significantly higher in autumn than in spring and summer, and in winter than in summer.

We tested whether apiary-level DWV-A prevalence (i.e. the number of positive vs. negative colonies per apiary) was affected by host species or season (modeled as a continuous trait) using Generalised linear mixed models (GLMMs) with binomial error distribution and logit link function, including the location of origin and the collection year as random effects. The minimum adequate model (MAM) was identified through model simplification using ANOVA and removal of non-significant terms. Measuring apiary-level prevalence per location and time-point, we found that both the host species (ANOVA, χ^2^ = 9.2649, *d.f*. = 1, *p* < 0.01) and the season in which samples were collected (ANOVA, χ^2^ = 9.7502, *d.f*. = 1, *p* < 0.01; modeled as a continuous variable) had a significant effect on prevalence, see Fig. 1. These factors did not significantly interact (ANOVA, χ^2^ = 0.2086, *d.f*. = 1, *p* = 0.65). We found that the region samples were collected in (North, Northeast, Northwest, East, Central and South China) did not significantly affect prevalence (ANOVA, χ^2^ = 7.2353, *d.f*. = 5, *p* = 0.2037). These results – significant effects of host species (ANOVA, χ^2^ = 11.469, *d.f*. = 1, *p* < 0.001) and season (ANOVA, χ^2^ = 19.399, *d.f*. = 3, *p* < 0.001) on prevalence, but no significant interaction (ANOVA, χ^2^ = 2.4559, *d.f*. = 3, *p* = 0.48) - were also replicated when modeling season as a discrete trait rather than a continuous trait in order to perform post-hoc tests, however for convergence reasons, year was not included as a random factor in these models. Prevalence was not only higher in *A. mellifera* than in *A. cerana* colonies (*z* = 2.827, *p* < 0.01), but was also found to be higher in autumn than in spring and summer (*z*_*spring*_ = −2.956, *p*_*spring*_ < 0.05; *z*_*summer*_ = −3.419, *p*_*spring*_ < 0.01) as well as higher in winter than summer (*z* = 2.913, *p* < 0.05) using multiple comparisons of means with Tukey contrasts.

Several previous reports include measures of DWV prevalence in one or both species in East Asia^35–42^ (see Table S3). Across all studies, the mean prevalence in *A. cerana* is 22% and 74% in *A. mellifera*. Testing the studies that measured prevalence in both species simultaneously, we find that prevalences are significantly different between species across all studies (glm with binomial error function, *z* = 7.22, *p* < 0.001).

### Phylogenetic and population genetic analysis

**Figure 2:**
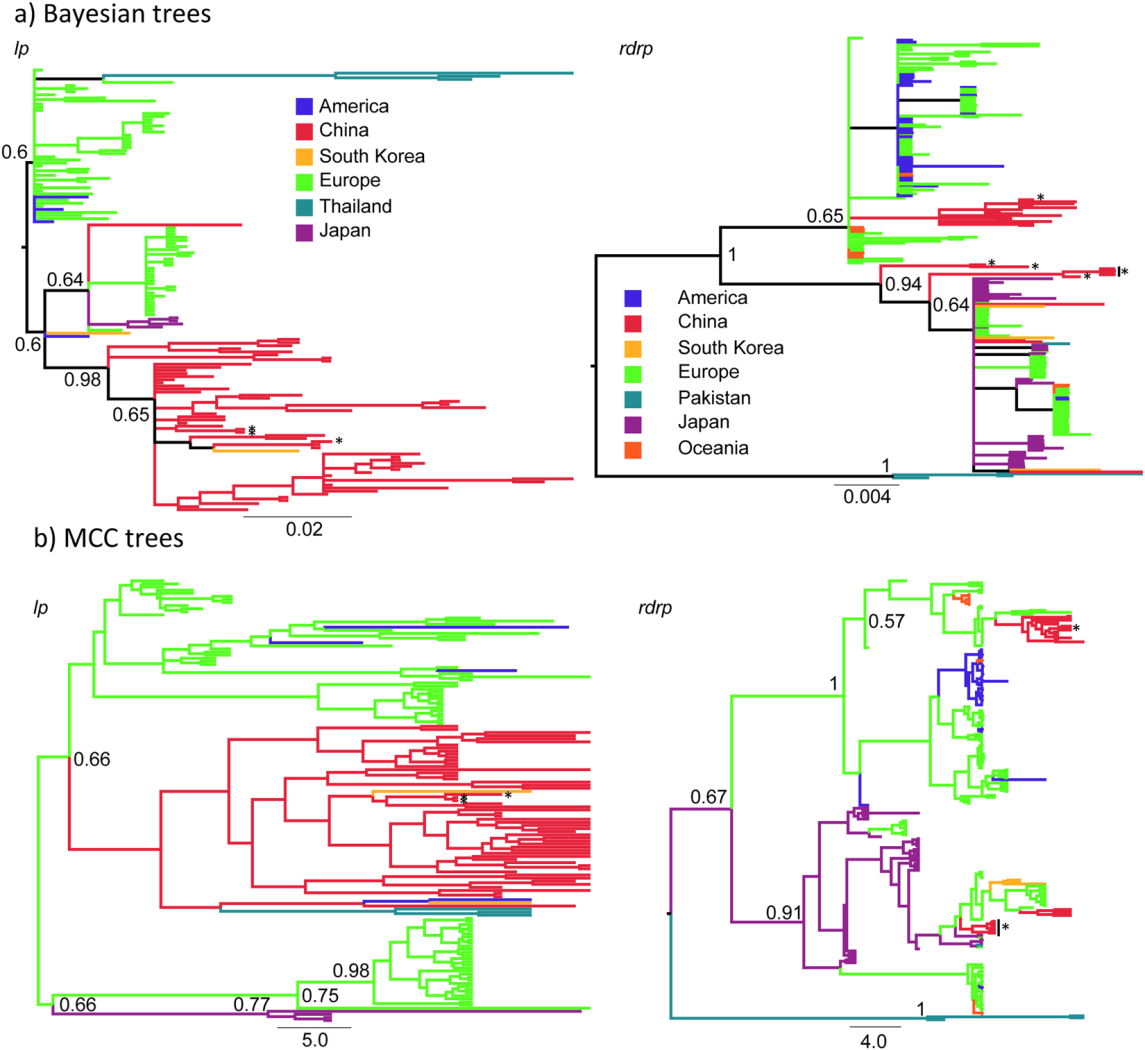
Phylogenetic trees for *lp*-, *rdrp*- and *vp3*-fragments; (**a**) Bayesian trees (**b**). MCC trees constructed with TreeAnnotator based on BEAST runs. Star symbols indicate samples isolated from *A. cerana* in China. All trees were midpoint-rooted. Posterior support (MCC trees) and Bayesian probabilities (Bayesian trees) above 0.5 are indicated up to the 3rd node from the midpoint root; horizontal bars indicate the time scale in years. In the MCC trees, the branches are colored according to the lineages’ inferred geographic origin. See supplementary information (Figs S5–S8) for enlarged tree figures including sample names/Genbank accession numbers.

We reconstructed the DWV-A phylogeny independently via three gene fragments *(lp-, rdrp-* and *vp3*), using all available comparative DWV-A sequences on Genbank (Fig. 2; see supplementary information for trees including Genbank accession numbers) from both *A. mellifera* and *A. cerana*. A Bayesian approach (Fig. 2a) showed that the *lp-* and *rdrp*-fragments produced relatively well resolved phylogenies, whereas the *vp3-*fragment contained too little genetic variation to resolve DWV-A population structure beyond the split between the globally distributed DWV-A isolates and divergent isolates from Pakistan^26^ (see Fig. S2). We therefore restricted further population genetic analysis to the phylogenetically informative *lp-* and *rdrp-*fragments. For the *rdrp-*fragment, Chinese samples fall in two distinct clades. For the *lp-*fragment on the other hand, the maximum clade credibility (MCC) tree shows Chinese and South Korean samples in a single well-supported clade; this result is also largely supported by the Bayesian phylogeny, which does not take geographic origin into account. While it is important to note that the alignments on which these phylogenetic trees are based contain different individual samples both globally and as collected for this study within China, several samples from the same Apiary (ZJ-1&2; HuB-5&7; XJ-4; JS-2; HeB-1&2; LN-5) are included for both gene fragments; in the *lp-*tree, these DWV sequences form a monophyletic clade, whereas the *rdrp-*tree shows that two viral sequences of different phylogentic origins are present in the same apiary.

We found no evidence for genetic differentiation between DWV sequences isolated from *A. cerana* or *A. mellifera* (*lp*-fragment: *N*_*A.cerana*_ = 3, *N*_*A.mellifera*_ = 127, *rdrp*-fragment: *N*_*A.cerana*_ = 8, *N*_*A.mellifera*_ = 184). As shown in Fig. 2, samples from Chinese *A. cerana* apiaries are nested within other samples isolated from China. A group of 7 isolates from *A. cerana* in the *rdrp-*fragment (JX679473-JX679480) cluster together (see black vertical bar on *rdrp*-trees in Fig. 2); however, these samples from Yunnan province come from a single Genbank submission that did not include corresponding samples from *A. mellifera* and lend no support for genetic differentiation between DWV-A populations in *A. cerana* and *A. mellifera* without further investigation. All subsequent analysis combine DWV sequences from both host species.

The different phylogenies for the Chinese isolates seen in the two fragments (Fig. 2) are also reflected in their demographic parameters as reconstructed with the coalescent sampler BEAST^43^. Exponential population growth is supported for the Chinese samples within the *lp*-fragment (mean doubling time 7.66 years, 95% Highest Posterior Density (HPD) 4.53–20.75), while the 95% HPD for the exponential growth rate overlaps with 0 for the *rdrp-*fragment (95% HPD −0.03–0.23). An excess of low frequency polymorphism was detected in both fragments using a coalescent simulation of Tajima’s D in DNAsp v.5^44^ (*D*_*lp*_ = −1.69, *p*_*lp*_ < 0.05, 95% Confidence Interval −1.598–1.946; *D*_*rdrp*_ = −1.75, *p*_*rdrp*_ > 0.01, 95% Confidence Interval −1.565–1.855), also indicating potential population size expansion in the Chinese DWV population. For both fragments, the most recent common ancestor of the Chinese DWV-A population is reconstructed about 3 decades ago (*lp*-fragment: 33 years, HPD 21–48 years, *rdrp*-fragment: 31 years, HPD 20–45 years; root height: *lp*-fragment: 47 years, HPD 37–60 years, *rdrp*-fragment: 38 years, HPD 24–57 years).

Measuring population differentiation based on the proportion of between-population nucleotide differences^45^, we found moderate levels of population differentiation between China, East Asia (Japan and South Korea as well as, for the *lp*-fragment, Thailand), Europe and America (Kst_lp_ = 0.12, Kst_rdrp_ = 0.27, *p* < 0.001) in accordance with the phylogenetic trees. Across all three fragments, samples from China and East Asia (Japan, South Korea and, for the *lp*-fragment, Thailand) consistently showed the highest nucleotide diversities (see Table 1). Nucleotide diversity *π* was typically ~2–3 times higher for these populations than for the samples from Europe and America. Discrete trait analysis in BEAST, modelling the geographic origin as a discrete trait, however indicated that China is not a source population, but a sink population with Europe serving as a source for the Chinese DWV-A population (Bayes Factor = 17.14 and 499.84 for the *lp-* and *rdrp-*fragments respectively; see Tables S4, S5 for Bayes Factors between all populations). The phylogenetic trees (Fig. 2) indicate no geographic clustering within China based on the available genetic information. There is also no evidence for isolation by distance between samples from China based on geographic distance and Euclidian genetic distance (Mantel-test, 999 permutations, *lp*-fragment *p* = 0.51, *rdrp*-fragment *p* = 0.154).

**Table 1:** Nucleotide diversity *π* per fragment, number in bracket indicates sample size.

	*lp*	*rdrp*	*vp3*
China	0.057 [61]	0.027 [25]	0.021 [34]
East Asia	0.064 [69]	0.021 [40]	0.016 [10]
America	0.021 [4]	0.006 [36]	0.006 [27]
Europe	0.034 [67]	0.018 [109]	0.009 [41]

## Discussion

This multi-year survey of *A. mellifera* and *A. cerana* apiaries across China shows that, while DWV-A is genetically more diverse in China and East Asia than in the rest of the global populations sampled, China represents a sink population, rather than the source of this global epidemic. DWV is predominantly a disease of non-native European honey bees in China; DWV-A prevalence was significantly higher in *A. mellifera* (45.7% DWV-positive colonies) than in *A. cerana* (5.6%), with prevalences increasing at the end of the season. The emerging DWV-B was rare in *A. mellifera* and absent in *A. cerana*.

In combination with previous studies of DWV in *A. mellifera* and *A. cerana* in East Asia^35–42^, we can conclude that DWV is more prevalent in the European honey bee than in the Asian honey bee in East Asia. This lower prevalence may be due to a reduced exposure of Asian honey bees to DWV vectored by Varroa mites. *A. cerana* has co-evolved with Varroa ectoparasites; in contrast to *A. mellifera*, Asian honey bees can control mite populations by grooming of adult bees, removing infested brood and by effectively restricting Varroa reproduction to drone brood^27,28^.

The present study shows that there is no evidence for genetic differentiation between DWV isolates from *A. mellifera* and *A. cerana* (Fig. 2). This corroborates findings from other smaller studies from China^40,42,46^ and Japan^39^. There is thus no evidence that *A. cerana* harbours a distinct DWV-A-clade or acts as a reservoir of infection. To uncover the full viral flora of Asian honey bees, including testing whether these populations harbor divergent DWV strains and whether they may be the ancestral host of DWV pre-dating the global emergence of DWV-A in concert with the global spread of Varroa^26^, next-generation sequencing studies are needed^47^. Next-generation sequencing studies allow an unbiased de-novo approach to discover the true viral diversity in host species.

This study was carried out across two years, sampling throughout the year. This revealed strong seasonality (Fig. 1), with prevalence highest at the end of the season in autumn and winter. This matches patterns reported from studies in France^48^, Denmark^49^ and the United States^50^. Both Tentcheva *et al*.^48^ and Francis *et al*.^49^ report high and increasing prevalence in worker honey bees with the highest prevalence reported in autumn (the studies did not include workers collected in winter). Using a microarray, Runckel *et al*.^50^ found low prevalence of DWV with sporadic incidences only in autumn and winter. This seasonal change in prevalence, with reduced prevalence in spring and summer, may be caused by increased mortality of DWV-infected workers in the cold season. Natsopoulou *et al*.^25^ showed an increase in over-winter workforce mortality in colonies infected by DWV-B (note no DWV-A was detected in this field study). An increase in mortality of infected workers would lead to a reduction in the detected prevalence in spring. Additionally, seasonality in DWV prevalence could also be linked to seasonal population dynamics or behavioural changes in *V. destructor*, with an increase in phoretic mite density in adult bees typically reported in autumn^51^. Other factors that may contribute to this strong seasonality may be environmental stressors, such as forage availability and diversity (e.g.^52,53^), and a build-up of exposure to chemicals throughout the season^54^, which may lead to a trade-off in resistance to DWV via the bees’ immune system^55^.

Comparing Chinese and East Asian DWV populations to the global DWV data, it is evident that these populations harbor higher genetic diversity than other populations, especially the very well sampled European population (Table 1). Comparatively high genetic diversity is a potential indicator of an ancestral population, which could support two non-exclusive hypotheses that: a) these populations represent the ancestral source population of the current DWV-A population, spread in conjunction with its emergent vector *V. destructor*; b) the high genetic diversity is maintained or generated because of the diversity in Chinese honey bee species and their co-evolved ectoparasitic mite species^6,56^. In contrast, the Hawaiian Varroa invasion has demonstrated that the introduction of Varroa as a viral vector can lead to a drastic reduction in DWV’s genetic variation within *A. mellifera*^15^. However, a phylogenetic reconstruction using geographic origin as a discrete trait clearly shows China and neighboring South Korea to be sink rather than source populations for global DWV-A. The most recent common ancestors date within the last 30–50 years, several decades after the acquisition and spread of the Varroa mite^26^. Thus we find no support for the hypothesis that the current DWV-A epidemic has its origin in East Asian honey bee and Varroa populations. Instead, these results, in line with the global geographic reconstruction of the DWV-A epidemic^26^, point towards a non-Asian origin of the current epidemic, with Varroa serving as an amplifier rather than the ancestral source of the epidemic. The comparatively high genetic variation in China is compatible with a recent population expansion of the Chinese DWV-population; there is little evidence that the Chinese DWV populations show inherently higher evolutionary rates (sup Fig. S3, S4). The comparatively high genetic variation has a recent origin within the last 4 decades, not pre-dating the spread of non-native *A. mellifera* or its acquisition of Varroa as a viral vector in China^6^. Whether the higher genetic variation within Chinese DWV populations may relate to the higher species-level diversity of Chinese honey bees and their co-evolved mites will need to be addressed by surveys including not only *A. mellifera, A. cerana* and *V. destructor*, but also the wild species (*A. florea, A. dorsata, A. andreniformis, A. laboriosa*) and their respective mites^6,56^.

The DWV-A phylogeny within China also shows a potential for recombination and divergent evolution within its genome: whereas the Chinese isolates are part of a well-supported single clade in the *lp-*fragment, isolates are distributed over two well-supported clades for the *rdrp-*fragment. This pattern is indicative of recombination within the DWV genome. While the present study cannot directly show recombination within individuals, 6 isolates that are placed on different clades for the two fragments come from the same apiaries, which are likely to be genetically highly similar. This pattern is reminiscent of the DWV-A/B recombinants found in field studies in Israel^57^ and the UK^58,59^ and may indicate that recombination may play a role in DWV evolution not only between DWV-A and DWV-B^22^ but also within DWV-A itself.

DWV is a species complex consisting of at least 3 distinct subtypes^34^, with DWV-A being globally distributed^26^. While we find sequence evidence for the presence of DWV-B in China in this study, this subtype showed a prevalence of less than 1% and was not found in *A. cerana*. Previous studies that conducted phylogenetic analysis have only detected DWV-A in China^40,42,46^ and Japan^39^. However, it has to be noted that earlier studies used primers designed to amplify DWV-A and may thus have underestimated the prevalence of DWV-B^60^. Given the rapid population expansion of DWV-B in Europe^25,61^ and its recent emergence and spread in North America^22^, Chinese honey bees may be at the cusp of a DWV-B epidemic. This is of particular concern given the increased virulence of DWV-B in European honey bees^25,62^. In addition, this viral type, like DWV-A^24^, also shows potential spillover into bumblebees in the presence of Varroa^61^. This potential spread is of particular concern in China, which is a threatened hotspot for honey bee and bumble bee biodiversity^63^. The potential spread of DWV-B in China may allow testing whether competition between viral strains leads to a reduction in within-strain genetic diversity, an alternative hypothesis for the high genetic diversity found in Chinese DWV populations.

In conclusion, our results show Chinese honey bee populations are experiencing a DWV-A epidemic with exponential viral population expansion, but that this viral population is not a source of the current global epidemic^26^. We thus find no evidence that the globally spreading DWV-A strain has switched host from *A. cerana* to *A. mellifera* with its vector, the Varroa mite, nor that this currently globally dominant strain or the emerging DWV-B have originated in East Asia. This raises the questions of how the introduction of the non-native *A. mellifera* and the acquisition of Varroa as a viral vector in Asia have affected viral epidemiology in Asian wild and managed honey bee populations; addressing these questions calls for surveys of wild and managed populations using *de novo* next-generation sequencing approaches to uncover the full viral diversity in these populations. Our results show that the emerging virulent DWV-B^25,60,62,64^ is also present in Chinese *A. mellifera*. These findings call for urgent surveillance work on DWV-B in East Asia, to prevent and mitigate its further spread into apiculture and wild populations. The potential spread of DWV-B to China is a further demonstration that the unregulated movement of honey bee queens and colonies across borders and geographic regions needs to be curbed to prevent the spread of emerging pathogens, a risk which needs to the considered in the global movement of all live animal and plant material.

## Methods

### Samples

**Figure 3:**
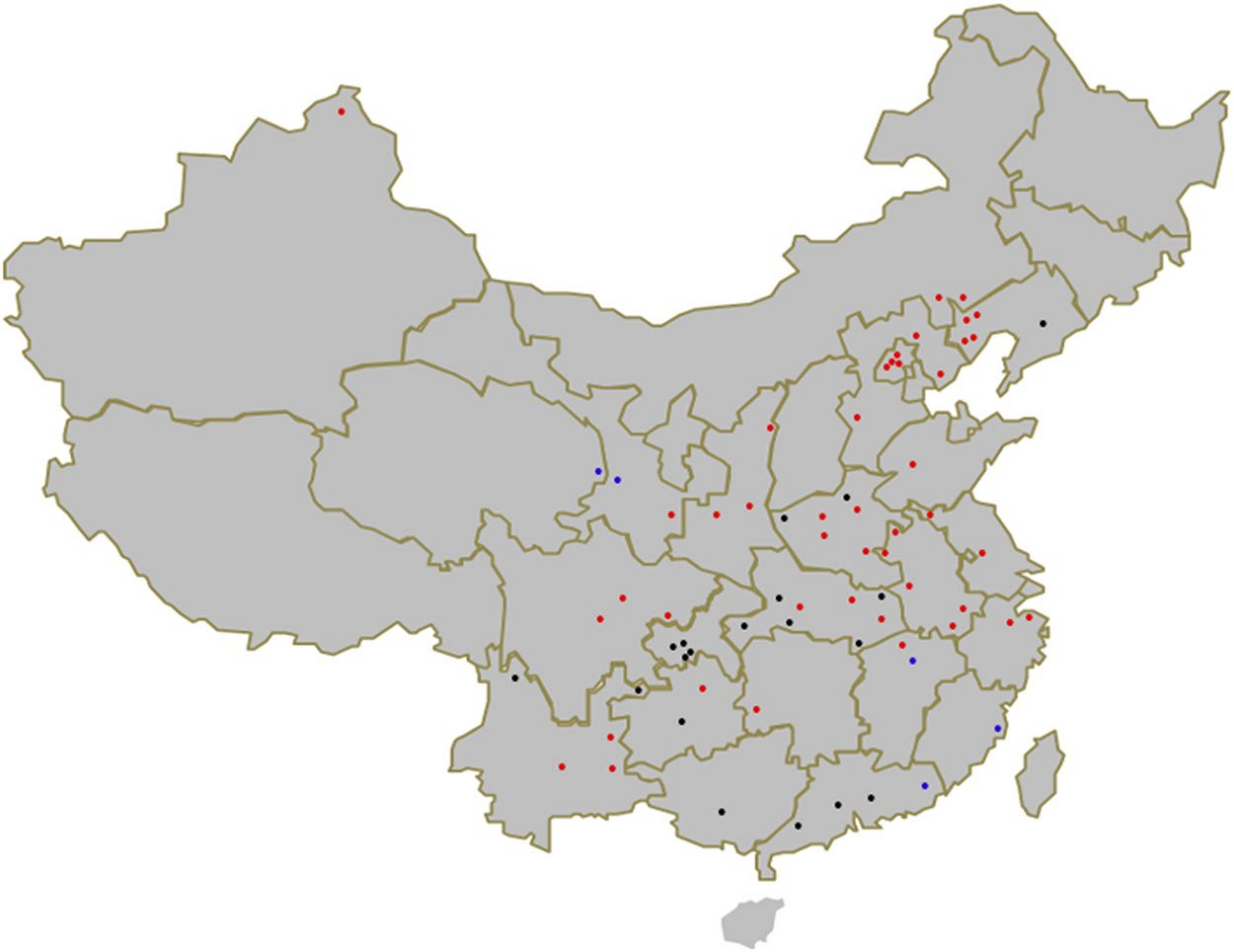
Sampling locations; *A. mellifera* sites are indicated in red, *A. cerana* in black and sites where both *A. mellifera* and *A. cerana* were collected are indicated in blue.

50 *A. cerana* and 117 *A. mellifera* hives across 22 Chinese provinces were sampled from March 2015 to February 2017, including Anhui, Jiangsu, Shandong, Zhejiang, Hebei, Beijing, Shannxi, Qinghai, Gansu, Xinjiang, Sichuan, Guizhou, Inner Mogolia, Chongqing, Hubei, Jiangxi, Henan, Liaoning, Guangdong, Fujian, Yunnan and Guangxi (Fig. 3; Table S1), with ~ fifty adult worker bees per hive being taken for further analysis.

Clinical signs of virus infection were not found in these colonies; thus, these colonies were considered to be capable of honey production, although they were reported either to have shown the presence of crawling bees or to have undergone important worker losses previously^65,66^. All of the above colonies were maintained consistent with guidelines for beekeeping practice and, in the case of *A. mellifera*, regularly monitored and treated with acaricides for mites. There was no effect on colony development reported due to mite infestation during the previous year. Living bee samples were placed in a small iron wire cage for direct transport or in 50 mL sterile tubes with dry ice for transport to the laboratory, where the samples were stored at −80 °C prior to use.

## RNA extraction

All individual samples were submerged in liquid nitrogen and ground into a fine powder using a pestle and mortar; this material was used in Trizol-based RNA extractions as described in Diao *et al*.^67^. The obtained RNA was dissolved in 20 µL of sterile water and stored at −80 °C prior to analysis. The quantity and purity of the RNA were measured using a Nanodrop spectrophotometer (Thermo Scientific, Beijing, China), with samples included for further analysis if OD 260/280 values were in the range of 1.8–2.0. The cDNA was synthesized using M-MLV reverse transcriptase with oligo dT primers according to the manufacturer’s instructions (Thermo, USA).

### RT-PCR amplification

Pooled samples of ~50 individuals were initially screened for DWV using RT-PCR according to standard procedures at the Institute of Apicultural Research. To specifically test for the presence of the two most common DWV strains, DWV-A and DWV-B, we used strain specific primers for DWV-A (DWV-F1a GGA AAC ATC TGG AAT TAG CGA CAA) and DWV-B (VDV-F1a GAA AAC ATT TGG AAT TAG CAA CGA C) respectively, with the conserved reverse primer DWV-VDV 7aR (AAT CCG TGA ATA TAG TGT GAG G)^68^. Only 2 out of 167 colonies were confirmed to be infected with DWV-B using specific primers and Sanger sequencing and we therefore focused further analysis on DWV-A.

Following the detection of positive hives via the pooled samples, DWV was amplified from 3 or 5 individuals per hive (see Table S1) using primers for three fragments (*lp*, *vp3* and *rdrp*)^26^ (see Table S6); these fragments were chosen based on the availability of comparative and informative sequence data on Genbank. The PCR products were sequenced by BGI (BGI Company, Shenzhen). The sequences of the *lp, vp3* and *rdrp-*fragments have been deposited in GenBank under accession numbers MF431915-431945 (*lp*-fragment), MF144195-144203, MF351971-351972 and MF431886-431914 (*vp3-*fragment) and MF667713-667747 (*rdrp-*fragment).

### Prevalence measures and statistical analysis

To take account of PCR assay efficiency and sensitivity (conservatively set at 95%)^32^, we used the R library epiR v.0.9–82^69^ and the function epi.prev to calculate the true prevalence with 95% confidence based on methods in Blaker^70^. We used R v. 3.5.3. in all analyses^71^. To test whether DWV-A prevalence at the apiary-level (the number of DWV-A-positive and negative hives in a location at a given sampling time-point) was affected by host species, and season, we used the lme4 package (v1.1-12)^72^ to run generalised linear mixed models (GLMMs) with binomial error distribution and a logit link function. The full model included the two-way interaction between the fixed effects host species (*A. cerana and A. mellifera*) and season (spring (March–May), summer (June–August), autumn (September–November) and winter (December–February) modeled either as a continuous or discrete trait). Location and year were included as random factors when modeling season as a continuous factor; year was excluded as a random factor when modeling season as a discrete trait to allow the model to converge. The minimum adequate model (MAM) was identified through removal of non-significant terms and comparison of models using ANOVA. We additionally tested whether the geographic origin by region (Center: Henan, Hubei, Jianxi; East: Anhui, Jiangsu, Shandong, Zhejiang; North: Beijing, Hebei; Northeast: Liaoning; Northwest: Chongqing, Gansu, Guizhou, Inner Mongolia, Qinghai, Sichuan, Xinjang; South: Guangdong, Guangxi, Yunnan) affected DWV-A prevalence, but due to a lack of convergence could not test for interactions with species and season. We used the glht function in the multicomp package v. 1.4–10^73^ for multiple comparisons of means using Tukey conrasts.

### Sequence analysis

Using BLAST^74^ and a cutoff of 90% sequence similarity, we identified all available comparative DWV-A sequences on Genbank which included country of origin, host organism and year of collection for phylogenetic analysis and created alignments in Geneious 6.1.8, optimizing the alignment length to increase the number of isolates from China. We constructed 3 alignments, *lp*-fragment (n = 143, 287 bp; 56 samples from China, including 14 new samples as well as isolates MF036686 and samples from Zhejiang (HG779848-9; 50; 52; 56-8; 60-61), Hainan (JF346640-54) and Shaanxi provinces (MF134371-83)); *vp3*-fragment (n = 135, 273 bp; 34 samples from China, including 20 new samples as well as MF036686 and samples from Shaanxi province (MF134371-83)); and *rdrp*-fragment (n = 224, 252 bp; 25 samples from China including MF036686 and samples from Yunnan province (JX679473-80)). We confirmed that there was no recombination within fragments at a *p*-value of 0.05, using the GENECONV^75^, MaxChi^76^, BootScan^77^ and SiScan^78^ algorithms in the Rdp4 package (v4.56)^77^.

### Phylogenetic reconstruction and population genetics

Beast 1.8^43^ was used for phylogenetic reconstruction with tip dates, with discrete trait models with asymmetric substitution models for geographic location; the sample size and genetic variation contained in the alignments was not sufficient to include host species as a trait. Jmodeltest (v.2.1)^79,80^ was used to determine suitable substitution models for each fragment based on the Bayesian Information Criterion (*lp-*fragment: TrN + G; *vp3*- and *rdrp-*fragments HKY + G) and substitution rates were partioned between the 1^st^ & 2^nd^ and 3^rd^ codon positions. The evolutionary rate prior was set for each fragment according to Wilfert *et al*.^26^ (*lp*-fragment: normal prior with a mean of 0.91 × 10^−3^ (stdev = 0.24 × 10^−3^); *vp3*-fragment: normal prior with a mean of 1.85 × 10^−3^ (stdev = 0.35 × 10^−3^); *rdrp*-fragment: lognormal prior with a mean in real space of 1.28 × 10^−3^ (log(stdev) = 0.325)). Demographic and molecular clock rates were set according to model comparison via the path sampling maximum likelihood estimator; exponential growth was preferred for all fragments, with an exponential relaxed clock for the *lp-* and *vp3-*fragment and a lognormal relaxed clock for the *rdrp*-fragment. All models were checked for convergence in Tracer (v1.6) and run long enough to obtain effective sample sizes > 200 for all parameters, with a 10% burn-in, sampling the chain at equal distances to obtain a total of 10,000 trees per analysis. We produced Maximum Clade Credibility (MCC) trees (TreeAnnotator (v1.8.4)) to infer host ancestral state probabilities. Well supported rates for migration routes between geographic regions were identified using a Bayes factors analysis (SPREAD v1.0.6^81^), with a Bayes Factor of 3 as a cut-off. Phylogenetic trees were also produced for each alignment using MrBayes 3.2.6.

Using DNASPv5.10.1^44^ we calculated Tajima’s D to test for an excess of rare polymorphisms. Additionally, we calculated Kst, a measure of population differentiation based on the proportion of between-population nucleotide differences^45^ between China, East Asia (South Korea and Japan), Europe and America as well as the nucleotide diversity π. To test for isolation by distance between samples collected for this study in China, we calculated individual Euclidean genetic distances using the package adegenet^82^ in R; we used individual-based distances as many locations were represented by single sequences. We tested for isolation by distance via a Mantel test implemented following Legendre and Legendre^83^ in the R package vegan^84^ of the correlation between geographic distance and Euclidean genetic distance using 999 permutations.

## Supplementary information

Supplementary Information

## Data Availability

Sequence data have been deposited in GenBank under accession numbers MF431915-431945 (*lp*-fragment), MF144195-144203, MF351971-351972 and MF431886-431914 (*vp3-*fragment) and MF667713-667747 (*rdrp-*fragment).
