# Supplementary material for: Prevalence and population genetics of the emerging honey bee pathogen DWV in Chinese apiculture
Source: Sci Rep. 2019 Aug 19;9:12042. doi: 10.1038/s41598-019-48618-y (PMC6700284; doi:10.1038/s41598-019-48618-y)
Supplement: Supplementary file 1 — Supplementary Information [file 41598_2019_48618_MOESM1_ESM.pdf]

Prevalence and population genetics of the emerging honey bee pathogen DWV in Chinese apiculture

Qingyun Diao[1,2#], Dahe Yang[1,2#], Hongxia Zhao[3], Shuai Deng[1,2], Xinling Wang[1,2], Chunsheng Hou[1,2*], Lena Wilfert[4,5*]

[1]Institute of Apicultural Research, Chinese Academy of Agricultural Sciences, Beijing 100093, P.R. China
[2]Key Laboratory of Pollinating Insect Biology, Ministry of Agriculture, Beijing 100093, P.R. China
[3]Guangdong Key Laboratory of Animal Conservation and Resource Utilization, Guangdong Public Laboratory of Wild Animal Conservation and Utilization, Guangdong Institute of Applied Biological Resources, Guangzhou 510260, P.R. China
[4]Centre for Ecology and Conservation, University of Exeter, Penryn Campus, UK
[5]Institute of Evolutionary Ecology and Conservation Genomics, University of Ulm, 89079 Ulm, Germany

Supplementary Information

Table. S1: Apiary level data. Data aggregated at apiary-level for generalized mixed linear model analysis of DWV prevalence. Region: CC = Central China, EC = Eastern China, NC = North China, NEC = Northeastern China, NWC = Northwestern China, SC = Southern China; sample size N refers to number of colonies.

Apiary	location	Province	Region	date	host species	N positive	N negative	N colonies
Anhui-1	Bo Zhou	Anhui	EC	05.11.16	*A. mellifera*	1	0	1
Anhui-2	Huang shan	Anhui	EC	25.04.15	*A. mellifera*	0	2	2
Anhui-3	Huang shan	Anhui	EC	25.09.16	*A. mellifera*	1	1	2
Anhui-4	Jing de	Anhui	EC	01.12.16	*A. mellifera*	0	1	1
Anhui-5	Ling quan	Anhui	EC	29.11.16	*A. mellifera*	1	0	1
Anhui-6	Liu an	Anhui	EC	30.11.16	*A. mellifera*	1	1	2
Beijing-1	Chang ping	Beijing	NC	07.12.16	*A. mellifera*	1	0	1
Beijing-2	Chao yang	Beijing	NC	07.12.16	*A. mellifera*	1	0	1
BeiJing-3	Meng tou gou	BeiJing	NC	24.10.15	*A. mellifera*	0	2	2
BeiJing-4	Xiang shan	BeiJing	NC	20.04.15	*A. mellifera*	0	2	2
Chongqing-1	Jiu long	Chongqing	NWC	18.08.15	*A. cerana*	1	2	3
Chongqing-2	Nan chuan	Chongqing	NWC	18.08.15	*A. cerana*	0	5	5
Chongqing-3	Pu ling	Chongqing	NWC	18.08.15	*A. cerana*	0	2	2
Chongqing-4	Wan sheng	Chongqing	NWC	18.08.15	*A. cerana*	0	2	2
Chongqing-5	Yong tang	Chongqing	NWC	18.08.15	*A. cerana*	0	3	3
Fujian-1	Fu zhou	Fujian	SC	25.11.15	*A. cerana*	1	2	3
Gansu-1	Hong gu	Gansu	NWC	05.09.16	*A. cerana*	2	1	3
Gansu-2	Tian shui	Gansu	NWC	09.11.15	*A. mellifera*	1	1	2
Guangdong-1	Feng shun	Guangdong	SC	17.11.16	*A. mellifera*	1	1	2
Guangdong-2	Fo shan	Guangdong	SC	17.11.16	*A. cerana*	0	1	1
Guangdong-3	Hui zhou	Guangdong	SC	28.04.15	*A. cerana*	0	1	1
Guangdong-4	Luo ding	Guangdong	SC	28.04.15	*A. cerana*	0	2	2
Guangxi-1	Nan ning	Guangxi	SC	08.12.16	*A. cerana*	0	1	1
Guizhou-1	Gui yang	Guizhou	NWC	25.09.16	*A. cerana*	0	2	2
Guizhou-2	Mei tan	Guizhou	NWC	31.10.16	*A. mellifera*	1	1	2
Hebei-1	Ling shou	Hebei	NC	07.12.16	*A. mellifera*	2	0	2
Hebei-2	Luan ping	Hebei	NC	19.11.15	*A. mellifera*	0	1	1
Hebei-3	Tang shan	Hebei	NC	08.06.16	*A. mellifera*	0	3	3
Henan-1	Ping yu	Henan	CC	09.04.15	*A. mellifera*	0	5	5
Henan-2	San men xia	Henan	CC	13.06.15	*A. cerana*	0	2	2
Henan-3	Xin cheng	Henan	CC	24.10.15	*A. mellifera*	1	0	1
Henan-4	Xin xiang	Henan	CC	13.06.15	*A. cerana*	0	2	2
Hubei-1	An lu	Hubei	CC	04.12.16	*A. mellifera*	1	0	1
Hubei-2	Dang yang	Hubei	CC	30.11.16	*A. mellifera*	11	1	12
Hubei-3	E zhou	Hubei	CC	19.01.17	*A. mellifera*	1	1	2
Hubei-4	En shi	Hubei	CC	19.04.15	*A. cerana*	0	1	1
Hubei-5	Ma cheng	Hubei	CC	19.04.15	*A. cerana*	0	4	4
Hubei-6	Shen nong jia	Hubei	CC	19.04.15	*A. cerana*	0	1	1
Hubei-7	Tong cheng	Hubei	CC	19.04.15	*A. cerana*	0	1	1

Hubei-8	Yi dou	Hubei	CC	19.04.15	*A. cerana*	0	1	1
Hubei-9	Zhong ze	Hubei	CC	13.03.17	*A. mellifera*	0	1	1
Hunan-1	Huai hua	Hunan	CC	02.03.17	*A. mellifera*	0	1	1
Inner Mogolia-1	Ao han	Inner Mogolia	NWC	28.11.15	*A. mellifera*	0	1	1
Inner Mogolia-2	Ao han	Inner Mogolia	NWC	20.06.15	*A. mellifera*	0	2	2
Inner Mogolia-3	Ao han	Inner Mogolia	NWC	27.06.15	*A. mellifera*	0	2	2
Inner Mogolia-4	Ao han	Inner Mogolia	NWC	30.06.15	*A. mellifera*	0	2	2
Inner Mogolia-5	Ao han	Inner Mogolia	NWC	30.07.15	*A. mellifera*	0	2	2
Inner Mogolia-6	Ao han	Inner Mogolia	NWC	30.12.15	*A. mellifera*	1	0	1
Inner Mogolia-7	Aohai	Inner Mogolia	NWC	20.06.15	*A. mellifera*	1	0	1
Inner Mogolia-8	Aohai	Inner Mogolia	NWC	28.08.15	*A. mellifera*	1	0	1
Inner Mogolia-9	Chi feng	Inner Mogolia	NWC	30.06.15	*A. mellifera*	0	1	1
Inner Mogolia-10	Chi feng	Inner Mogolia	NWC	30.12.15	*A. mellifera*	0	1	1
Jiangsu-1	Xu zhou	Jiangsu	EC	27.08.15	*A. mellifera*	0	1	1
Jiangsu-2	Yang zhou	Jiangsu	EC	14.11.16	*A. mellifera*	1	0	1
Jiangsu-3	Yang zhou	Jiangsu	EC	11.04.16	*A. mellifera*	2	3	5
Jiangxi-1	Jiu jiang	Jiangxi	CC	27.02.17	*A. mellifera*	0	1	1
Jiangxi-2	Nan chang	Jiangxi	CC	28.09.16	*A. mellifera*	0	1	1
Jiangxi-3	Nan chang	Jiangxi	CC	01.11.16	*A. cerana*	1	1	2
Liaoning-1	Bei piao	Liaoning	NEC	30.06.15	*A. mellifera*	0	2	2
Liaoning-2	Bein xi	Liaoning	NEC	28.08.15	*A. cerana*	0	3	3
Liaoning-3	Chao yang	Liaoning	NEC	16.11.15	*A. mellifera*	0	1	1
Liaoning-4	Chao yang	Liaoning	NEC	05.11.15	*A. mellifera*	1	1	2
Liaoning-5	Chao yang	Liaoning	NEC	18.05.15	*A. mellifera*	1	0	1
Liaoning-6	Chao yang	Liaoning	NEC	28.12.15	*A. mellifera*	1	0	1
Liaoning-7	Hu lu dao	Liaoning	NEC	29.07.15	*A. mellifera*	0	2	2
Liaoning-8	Hu lu dao	Liaoning	NEC	28.12.15	*A. mellifera*	1	0	1
Liaoning-9	Jian chang	Liaoning	NEC	01.11.16	*A. mellifera*	1	0	1
Qinghai-1	Min he	Qinghai	NWC	05.09.16	*A. cerana*	1	3	4
Qinghai-2	Min he	Qinghai	NWC	05.09.16	*A. mellifera*	2	0	2
Shandong-1	Ji nan	Shandong	EC	14.11.16	*A. mellifera*	1	0	1
Shannxi-1	Fu ping	Shannxi	NWC	11.11.15	*A. mellifera*	0	1	1
Shannxi-2	Qian xian	Shannxi	NWC	05.09.16	*A. mellifera*	1	1	2
Shannxi-3	Wu bao	Shannxi	NWC	28.11.16	*A. mellifera*	2	1	3
Sichuan-1	An yue	Sichuan	NWC	08.03.17	*A. mellifera*	0	1	1
Sichuan-2	Cheng du	Sichuan	NWC	05.11.15	*A. mellifera*	0	1	1
Sichuan-3	Cheng du	Sichuan	NWC	01.10.15	*A. mellifera*	1	0	1
Sichuan-4	E mei shan	Sichuan	NWC	16.01.17	*A. mellifera*	1	1	2
Xinjiang-1	Yi li	Xinjiang	NWC	27.09.16	*A. mellifera*	4	5	9
Yunnan-1	Qu jing	Yunnan	SC	12.12.16	*A. mellifera*	0	1	1
Yunnan-2	Ta cheng	Yunnan	SC	14.10.15	*A. cerana*	0	1	1
Yunnan-3	Wei xi	Yunnan	SC	12.10.15	*A. cerana*	0	1	1
Yunnan-4	Yi men	Yunnan	SC	12.12.16	*A. mellifera*	0	1	1
Yunnan-5	Zhong shu	Yunnan	SC	12.12.16	*A. mellifera*	1	1	2
Zhejiang-1	Ci xi	Zhejiang	EC	25.05.16	*A. mellifera*	3	1	4
Zhejiang-2	Shao xing	Zhejiang	EC	07.12.15	*A. mellifera*	1	0	1

Table S2. Colony-level sample information. The colony name refers to the sequence name. The number (N) of screened individuals refers to the number of individuals used for further analysis after screening pools of ~50 individuals per hive; NA indicates that the pool tested negative.

Colony	Apiary	location	Province	Region	collection date	host species	DWV positive	N screened individuals
AH-1	Anhui-6	Liu an	Anhui	EC	30.11.16	*A. mellifera*	yes	3
AH-2	Anhui-5	Ling quan	Anhui	EC	29.11.16	*A. mellifera*	yes	3
AH-3	Anhui-3	Huang shan	Anhui	EC	25.9.16	*A. mellifera*	yes	3
AH-4	Anhui-1	Bo Zhou	Anhui	EC	5.11.16	*A. mellifera*	yes	3
AH-5	Anhui-3	Huang shan	Anhui	EC	25.9.16	*A. mellifera*	no	NA
AH-6	Anhui-4	Jing de	Anhui	EC	1.12.16	*A. mellifera*	no	NA
AH-7	Anhui-6	Liu an	Anhui	EC	30.11.16	*A. mellifera*	no	NA
AH-8	Anhui-2	Huang shan	Anhui	EC	25.4.15	*A. mellifera*	no	NA
AH-9	Anhui-2	Huang shan	Anhui	EC	25.4.15	*A. mellifera*	no	NA
BJ-1	Beijing-2	Chao yang	Beijing	NC	7.12.16	*A. mellifera*	yes	3
BJ-2	Beijing-1	Chang ping	Beijing	NC	7.12.16	*A. mellifera*	yes	3
BJ-3	BeiJing-3	Meng tou gou	BeiJing	NC	24.10.15	*A. mellifera*	no	NA
BJ-4	BeiJing-3	Meng tou gou	BeiJing	NC	24.10.15	*A. mellifera*	no	NA
BJ-5	BeiJing-4	Xiang shan	BeiJing	NC	20.4.15	*A. mellifera*	no	NA
BJ-6	BeiJing-4	Xiang shan	BeiJing	NC	20.4.15	*A. mellifera*	no	NA
CQ-1	Chongqing-1	Jiu long	Chongqing	NWC	18.8.15	*A. cerana*	yes	3
CQ-2	Chongqing-2	Nan chuan	Chongqing	NWC	18.8.15	*A. cerana*	no	NA
CQ-3	Chongqing-2	Nan chuan	Chongqing	NWC	18.8.15	*A. cerana*	no	NA
CQ-4	Chongqing-2	Nan chuan	Chongqing	NWC	18.8.15	*A. cerana*	no	NA
CQ-5	Chongqing-2	Nan chuan	Chongqing	NWC	18.8.15	*A. cerana*	no	NA
CQ-6	Chongqing-2	Nan chuan	Chongqing	NWC	18.8.15	*A. cerana*	no	NA
CQ-7	Chongqing-4	Wan sheng	Chongqing	NWC	18.8.15	*A. cerana*	no	NA
CQ-8	Chongqing-4	Wan sheng	Chongqing	NWC	18.8.15	*A. cerana*	no	NA
CQ-9	Chongqing-1	Jiu long	Chongqing	NWC	18.8.15	*A. cerana*	no	NA
CQ-10	Chongqing-1	Jiu long	Chongqing	NWC	18.8.15	*A. cerana*	no	NA
CQ-11	Chongqing-3	Pu ling	Chongqing	NWC	18.8.15	*A. cerana*	no	NA
CQ-12	Chongqing-3	Pu ling	Chongqing	NWC	18.8.15	*A. cerana*	no	NA
CQ-13	Chongqing-5	Yong tang	Chongqing	NWC	18.8.15	*A. cerana*	no	NA
CQ-14	Chongqing-5	Yong tang	Chongqing	NWC	18.8.15	*A. cerana*	no	NA
CQ-15	Chongqing-5	Yong tang	Chongqing	NWC	18.8.15	*A. cerana*	no	NA
FJ-1	Fujian-1	Fu zhou	Fujian	SC	25.11.15	*A. cerana*	yes	3
FJ-2	Fujian-1	Fu zhou	Fujian	SC	25.11.15	*A. cerana*	no	NA
FJ-3	Fujian-1	Fu zhou	Fujian	SC	25.11.15	*A. cerana*	no	NA
GD-1	Guangdong-1	Feng shun	Guangdong	SC	17.11.15	*A. mellifera*	yes	3
GD-2	Guangdong-1	Feng shun	Guangdong	SC	17.11.16	*A. cerana*	no	NA
GD-3	Guangdong-2	Fo shan	Guangdong	SC	17.11.16	*A. cerana*	no	NA
GD-4	Guangdong-3	Hui zhou	Guangdong	SC	28.4.15	*A. cerana*	no	NA
GD-5	Guangdong-4	Luo ding	Guangdong	SC	28.4.15	*A. cerana*	no	NA
GD-6	Guangdong-4	Luo ding	Guangdong	SC	28.4.15	*A. cerana*	no	NA
GD-7	Guangxi-1	Nan ning	Guangxi	SC	8.12.16	*A. cerana*	no	Na
GS-1	Gansu-1	Hong gu	Gansu	NWC	5.9.16	*A. cerana*	yes	3
GS-2	Gansu-1	Hong gu	Gansu	NWC	5.9.16	*A. mellifera*	yes	3

GS-3	Gansu-2	Tian shui	Gansu	NWC	9.11.15	*A. mellifera*	yes	3
GS-4	Gansu-1	Hong gu	Gansu	NWC	5.9.16	*A. mellifera*	no	NA
GS-5	Gansu-2	Tian shui	Gansu	NWC	9.11.15	*A. mellifera*	no	NA
GZ-1	Guizhou-2	Mei tan	Guizhou	NWC	31.10.16	*A. mellifera*	yes	5
GZ-2	Guizhou-1	Gui yang	Guizhou	NWC	25.9.16	*A. cerana*	no	NA
GZ-3	Guizhou-1	Gui yang	Guizhou	NWC	25.9.16	*A. cerana*	no	NA
GZ-4	Guizhou-2	Mei tan	Guizhou	NWC	31.10.16	*A. mellifera*	no	NA
HeB-1	Hebei-1	Ling shou	Hebei	NC	7.12.16	*A. mellifera*	yes	3
HeB-2	Hebei-1	Ling shou	Hebei	NC	7.12.16	*A. mellifera*	yes	3
HeB-3	Hebei-2	Luan ping	Hebei	NC	19.11.15	*A. mellifera*	no	NA
HeB-4	Hebei-3	Tang shan	Hebei	NC	8.6.16	*A. mellifera*	no	NA
HeB-5	Hebei-3	Tang shan	Hebei	NC	8.6.16	*A. mellifera*	no	NA
HeB-6	Hebei-3	Tang shan	Hebei	NC	8.6.16	*A. mellifera*	no	NA
HN-2	Henan-3	Xin cheng	Henan	CC	24.10.15	*A. mellifera*	yes	3
HN-5	Henan-4	Xin xiang	Henan	CC	13.6.15	*A. cerana*	no	NA
HN-6	Henan-4	Xin xiang	Henan	CC	13.6.15	*A. cerana*	no	NA
HN-7	Henan-1	Ping yu	Henan	CC	9.4.15	*A. mellifera*	no	NA
HN-8	Henan-1	Ping yu	Henan	CC	9.4.15	*A. mellifera*	no	NA
HN-9	Henan-1	Ping yu	Henan	CC	9.4.15	*A. mellifera*	no	NA
HN-10	Henan-1	Ping yu	Henan	CC	9.4.15	*A. mellifera*	no	NA
HN-11	Henan-1	Ping yu	Henan	CC	9.4.15	*A. mellifera*	no	NA
HN-12	Henan-2	San men xia	Henan	CC	13.6.15	*A. cerana*	no	NA
HN-13	Henan-2	San men xia	Henan	CC	13.6.15	*A. cerana*	no	NA
HuB-1	Hubei-2	Dang yang	Hubei	CC	30.11.16	*A. mellifera*	yes	5
HuB-2	Hubei-3	E zhou	Hubei	CC	19.1.17	*A. mellifera*	yes	5
HuB-3	Hubei-1	An lu	Hubei	CC	4.12.16	*A. mellifera*	yes	3
HuB-4	Hubei-2	Dang yang	Hubei	CC	30.11.16	*A. mellifera*	yes	5
HuB-5	Hubei-2	Dang yang	Hubei	CC	30.11.16	*A. mellifera*	yes	5
HuB-6	Hubei-2	Dang yang	Hubei	CC	29.11.16	*A. mellifera*	yes	5
HuB-7	Hubei-2	Dang yang	Hubei	CC	30.11.16	*A. mellifera*	yes	5
HuB-8	Hubei-2	Dang yang	Hubei	CC	30.11.16	*A. mellifera*	yes	5
HuB-9	Hubei-2	Dang yang	Hubei	CC	30.11.16	*A. mellifera*	yes	5
HuB-10	Hubei-2	Dang yang	Hubei	CC	30.11.16	*A. mellifera*	yes	5
HuB-11	Hubei-2	Dang yang	Hubei	CC	30.11.16	*A. mellifera*	yes	5
HuB-12	Hubei-2	Dang yang	Hubei	CC	30.11.16	*A. mellifera*	yes	5
HuB-13	Hubei-2	Dang yang	Hubei	CC	30.11.16	*A. mellifera*	yes	5
HuB-14	Hubei-2	Dang yang	Hubei	CC	29.11.16	*A. mellifera*	no	NA
HuB-15	Hubei-4	En shi	Hubei	CC	19.4.15	*A. cerana*	no	NA
HuB-16	Hubei-3	E zhou	Hubei	CC	19.01.17	*A. mellifera*	no	NA
HuB-17	Hubei-5	Ma cheng	Hubei	CC	19.4.15	*A. cerana*	no	NA
HuB-18	Hubei-5	Ma cheng	Hubei	CC	19.4.15	*A. cerana*	no	NA
HuB-19	Hubei-5	Ma cheng	Hubei	CC	19.4.15	*A. cerana*	no	NA
HuB-20	Hubei-5	Ma cheng	Hubei	CC	19.4.15	*A. cerana*	no	NA
HuB-21	Hubei-6	Shen nong jia	Hubei	CC	19.4.15	*A. cerana*	no	NA
HuB-22	Hubei-7	Tong cheng	Hubei	CC	19.4.15	*A. cerana*	no	NA
HuB-23	Hubei-8	Yi dou	Hubei	CC	19.4.15	*A. cerana*	no	NA
HuB-24	Hubei-9	Zhong ze	Hubei	CC	13.3.17	*A. mellifera*	no	NA

HuB-25	Hunan-1	Huai hua	Hunan	CC	2.3.17	*A. mellifera*	no	NA
IM-1	Inner Mogolia-6	Ao han	Inner Mogolia	NWC	30.12.15	*A. mellifera*	yes	5
IM-2	Inner Mogolia-8	Aohai	Inner Mogolia	NWC	28.8.15	*A. mellifera*	yes	3
IM-3	Inner Mogolia-7	Aohai	Inner Mogolia	NWC	20.6.15	*A. mellifera*	yes	3
IM-4	Inner Mogolia-1	Ao han	Inner Mogolia	NWC	28.11.15	*A. mellifera*	no	NA
IM-5	Inner Mogolia-2	Ao han	Inner Mogolia	NWC	20.6.15	*A. mellifera*	no	NA
IM-6	Inner Mogolia-2	Ao han	Inner Mogolia	NWC	20.6.15	*A. mellifera*	no	NA
IM-7	Inner Mogolia-3	Ao han	Inner Mogolia	NWC	27.6.15	*A. mellifera*	no	NA
IM-8	Inner Mogolia-3	Ao han	Inner Mogolia	NWC	27.6.15	*A. mellifera*	no	NA
IM-9	Inner Mogolia-4	Ao han	Inner Mogolia	NWC	30.6.15	*A. mellifera*	no	NA
IM-10	Inner Mogolia-4	Ao han	Inner Mogolia	NWC	30.6.15	*A. mellifera*	no	NA
IM-11	Inner Mogolia-5	Ao han	Inner Mogolia	NWC	30.7.15	*A. mellifera*	no	NA
IM-12	Inner Mogolia-5	Ao han	Inner Mogolia	NWC	30.7.15	*A. mellifera*	no	NA
IM-13	Inner Mogolia-10	Chi feng	Inner Mogolia	NWC	30.12.15	*A. mellifera*	no	NA
IM-14	Inner Mogolia-9	Chi feng	Inner Mogolia	NWC	30.6.15	*A. mellifera*	no	NA
JS-1	Jiangsu-3	Yang zhou	Jiangsu	EC	11.4.16	*A. mellifera*	yes	3
JS-2	Jiangsu-2	Yang zhou	Jiangsu	EC	14.11.16	*A. mellifera*	yes	5
JS-3	Jiangsu-3	Yang zhou	Jiangsu	EC	9.4.16	*A. mellifera*	yes	5
JS-4	Jiangsu-3	Yang zhou	Jiangsu	EC	9.4.16	*A. mellifera*	no	NA
JS-5	Jiangsu-3	Yang zhou	Jiangsu	EC	9.4.16	*A. mellifera*	no	NA
JS-6	Jiangsu-3	Yang zhou	Jiangsu	EC	9.4.16	*A. mellifera*	no	NA
JS-7	Jiangsu-1	Xu zhou	Jiangsu	EC	27.8.15	*A. mellifera*	no	NA
JX-1	Jiangxi-3	Nan chang	Jiangxi	CC	1.11.16	*A. cerana*	yes	5
JX-2	Jiangxi-1	Jiu jiang	Jiangxi	CC	27.2.17	*A. mellifera*	no	NA
JX-3	Jiangxi-2	Nan chang	Jiangxi	CC	28.9.16	*A. mellifera*	no	NA
JX-4	Jiangxi-3	Nan chang	Jiangxi	CC	1.11.16	*A. cerana*	no	NA
LN-1	Liaoning-4	Chao yang	Liaoning	NEC	5.11.15	*A. mellifera*	yes	5
LN-2	Liaoning-8	Hu lu dao	Liaoning	NEC	28.12.15	*A. mellifera*	yes	5
LN-3	Liaoning-5	Chao yang	Liaoning	NEC	18.5.15	*A. mellifera*	yes	5
LN-4	Liaoning-9	Jian chang	Liaoning	NEC	1.11.16	*A. mellifera*	yes	5
LN-5	Liaoning-6	Chao yang	Liaoning	NEC	28.12.15	*A. mellifera*	yes	5
LN-6	Liaoning-1	Bei piao	Liaoning	NEC	30.6.15	*A. mellifera*	no	NA
LN-7	Liaoning-1	Bei piao	Liaoning	NEC	30.6.15	*A. mellifera*	no	NA
LN-8	Liaoning-2	Bein xi	Liaoning	NEC	28.8.15	*A. cerana*	no	NA
LN-9	Liaoning-2	Bein xi	Liaoning	NEC	28.8.15	*A. cerana*	no	NA
LN-10	Liaoning-2	Bein xi	Liaoning	NEC	28.8.15	*A. cerana*	no	NA
LN-11	Liaoning-4	Chao yang	Liaoning	NEC	5.11.15	*A. mellifera*	no	NA
LN-12	Liaoning-3	Chao yang	Liaoning	NEC	16.11.15	*A. mellifera*	no	NA
LN-13	Liaoning-7	Hu lu dao	Liaoning	NEC	29.7.15	*A. mellifera*	no	NA
LN-14	Liaoning-7	Hu lu dao	Liaoning	NEC	29.7.15	*A. mellifera*	no	NA
QH-1	Qinghai-2	Min he	Qinghai	NWC	5.9.16	*A. mellifera*	yes	3
QH-2	Qinghai-2	Min he	Qinghai	NWC	5.9.16	*A. mellifera*	yes	3
QH-3	Qinghai-1	Min he	Qinghai	NWC	4.9.16	*A. cerana*	yes	3
QH-4	Qinghai-1	Min he	Qinghai	NWC	5.9.16	*A. cerana*	no	NA
QH-5	Qinghai-1	Min he	Qinghai	NWC	5.9.16	*A. cerana*	no	NA
QH-6	Qinghai-1	Min he	Qinghai	NWC	5.9.16	*A. cerana*	no	NA
SC-1	Sichuan-4	E mei shan	Sichuan	NWC	16.1.17	*A. mellifera*	yes	5

SC-2	Sichuan-3	Cheng du	Sichuan	NWC	1.10.15	*A. mellifera*	yes	5
SC-3	Sichuan-1	An yue	Sichuan	NWC	8.3.17	*A. mellifera*	no	NA
SC-4	Sichuan-4	E mei shan	Sichuan	NWC	16.1.17	*A. mellifera*	no	NA
SC-5	Sichuan-2	Cheng du	Sichuan	NWC	5.11.15	*A. mellifera*	no	NA
SD-1	Shandong-1	Ji nan	Shandong	EC	14.11.16	*A. mellifera*	yes	3
SX-1	Shannxi-3	Wu bao	Shannxi	NWC	28.11.16	*A. mellifera*	yes	3
SX-2	Shannxi-2	Qian xian	Shannxi	NWC	5.9.16	*A. mellifera*	yes	3
SX-3	Shannxi-3	Wu bao	Shannxi	NWC	28.11.16	*A. mellifera*	yes	3
SX-4	Shannxi-1	Fu ping	Shannxi	NWC	11.11.15	*A. mellifera*	no	NA
SX-5	Shannxi-2	Qian xian	Shannxi	NWC	5.9.16	*A. mellifera*	no	NA
SX-6	Shannxi-3	Wu bao	Shannxi	NWC	28.11.16	*A. mellifera*	no	NA
XJ-1	Xinjiang-1	Yi li	Xinjiang	NWC	27.9.16	*A. mellifera*	yes	5
XJ-2	Xinjiang-1	Yi li	Xinjiang	NWC	27.9.16	*A. mellifera*	yes	5
XJ-3	Xinjiang-1	Yi li	Xinjiang	NWC	27.9.16	*A. mellifera*	yes	5
XJ-4	Xinjiang-1	Yi li	Xinjiang	NWC	27.9.16	*A. mellifera*	yes	5
XJ-5	Xinjiang-1	Yi li	Xinjiang	NWC	27.9.16	*A. mellifera*	no	NA
XJ-6	Xinjiang-1	Yi li	Xinjiang	NWC	27.9.16	*A. mellifera*	no	NA
XJ-7	Xinjiang-1	Yi li	Xinjiang	NWC	27.9.16	*A. mellifera*	no	NA
XJ-8	Xinjiang-1	Yi li	Xinjiang	NWC	27.9.16	*A. mellifera*	no	NA
XJ-9	Xinjiang-1	Yi li	Xinjiang	NWC	27.9.16	*A. mellifera*	no	NA
YN-1	Yunnan-5	Zhong shu	Yunnan	SC	12.12.16	*A. mellifera*	yes	3
YN-2	Yunnan-1	Qu jing	Yunnan	SC	12.12.16	*A. mellifera*	no	NA
YN-3	Yunnan-2	Ta cheng	Yunnan	SC	14.10.15	*A. cerana*	no	NA
YN-4	Yunnan-3	Wei xi	Yunnan	SC	12.10.15	*A. cerana*	no	NA
YN-5	Yunnan-4	Yi men	Yunnan	SC	12.12.16	*A. mellifera*	no	NA
YN-6	Yunnan-5	Zhong shu	Yunnan	SC	12.12.16	*A. mellifera*	no	NA
ZJ-1	Zhejiang-1	Ci xi	Zhejiang	EC	24.5.15	*A. mellifera*	yes	5
ZJ-2	Zhejiang-1	Ci xi	Zhejiang	EC	25.5.16	*A. mellifera*	yes	5
ZJ-3	Zhejiang-2	Shao xing	Zhejiang	EC	7.12.15	*A. mellifera*	yes	5
ZJ-4	Zhejiang-1	Ci xi	Zhejiang	EC	25.5.15	*A. mellifera*	yes	5
ZJ-5	Zhejiang-1	Ci xi	Zhejiang	EC	25.5.16	*A. mellifera*	no	NA

Table S3: DWV colony-level prevalence in *A. mellifera* and *A. cerana* reported in different studies from East Asia; prevalences were calculated based on the number of colonies assayed where prevalence measures were not directly presented. Reference numbers refer to the reference list contained within the supplementary material.

country	*A. mellifera*		*A. cerana*		reference
	prevalence	n (colonies)	prevalence	n (colonies)	
China	45.7	117	5.6	50	present study
China	45.5	33	9.5	42	[1]
China	94	170	64	70	[2]
China	74.8	103	78.7	47	[3]
China	74	46	73	37	[4]
China	NA		9	57	[5]
Japan	84	65	29	51	[6]
South Korea	NA		8.1	375	[7]
Vietnam	75	12	97	33	[4]
Vietnam	NA		3.3	180	[8]

Table S4. Bayes Factor for asymmetric BSSVS rates – *lp*-fragment. Bayes-factors below a cut-off of 3 are indicated by *.

	America	China	Europe	Japan	South Korea	Thailand
America	NA	*	*	*	4.27	*
China	*	NA	*	*	268.28	*
Europe	1596.97	17.14	NA	*	*	10.29
Japan	*	*	18.54	NA	*	*
South Korea	*	*	*	*	NA	*
Thailand	*	*	*	*	*	NA

Table S5: Asymmetric BSSVS rates – *rdrp*-fragment.

	America	China	Europe	Japan	Oceania	Pakistan	South Korea
America	NA	*	*	*	5.67	*	*
China	*	NA	*	*	*	*	*
Europe	47475.67	499.84	NA	*	120.67	*	22.9
Japan	*	*	4742.82	NA	*	108.4	*
Oceania	*	*	*	*	NA	*	*
Pakistan	*	*	*	*	*	NA	*
South Korea	*	*	*	*	*	*	NA

Table S6: Primers used for RT-PCR

Primer name	Sequence	Amplification conditions (35 cycles)	Length [bp]	Reference
Lp _F1153	ATTAAAAATGGCCTTTAGTTG	30 s at 94°C	653	[9]
Lp_ B1806	CTTTTCTAATTCAACTTCACC	30 s at 55°C		
		30 s at 72°C		
VP3_DWV F1	CCTGCTAATCAACAAGGACCTGG	30 s at 94°C	355	[10].
VP3_DWV B1	CAGAACCAATGTCTAACGCTAACCC	30 s at 55°C		
		30 s at 72°C		
RdRp_F15	TCCATCAGGTTCTCCAATAACGGA	30 s at 94°C	450	[11]
RdRp_B23	CCACCCAAATGCTAACTCTAAGCG	30 s at 49.2°C		
		30 s at 72°C		

Fig. S1: Bayesian phylogenetic tree of DWV-B including isolate SC-3 with Bayesian probabilities given in percent.

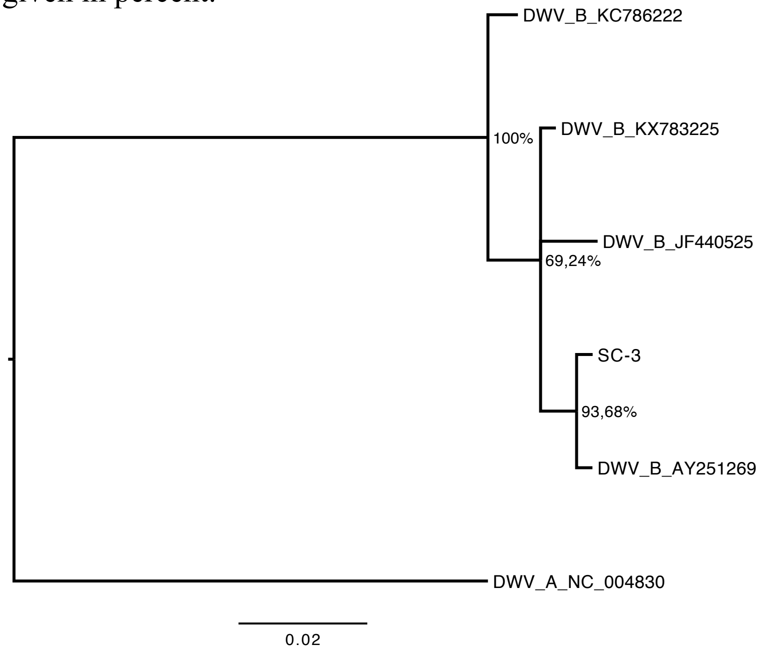

Fig. S2: Bayesian phylogenetic tree of the *vp3*-fragment. Star symbols indicate samples isolated from *A. cerana* in China. All trees were midpoint-rooted. Bayesian probabilities (Bayesian trees) above 0.5 are indicated up to the 3rd node from the midpoint root.

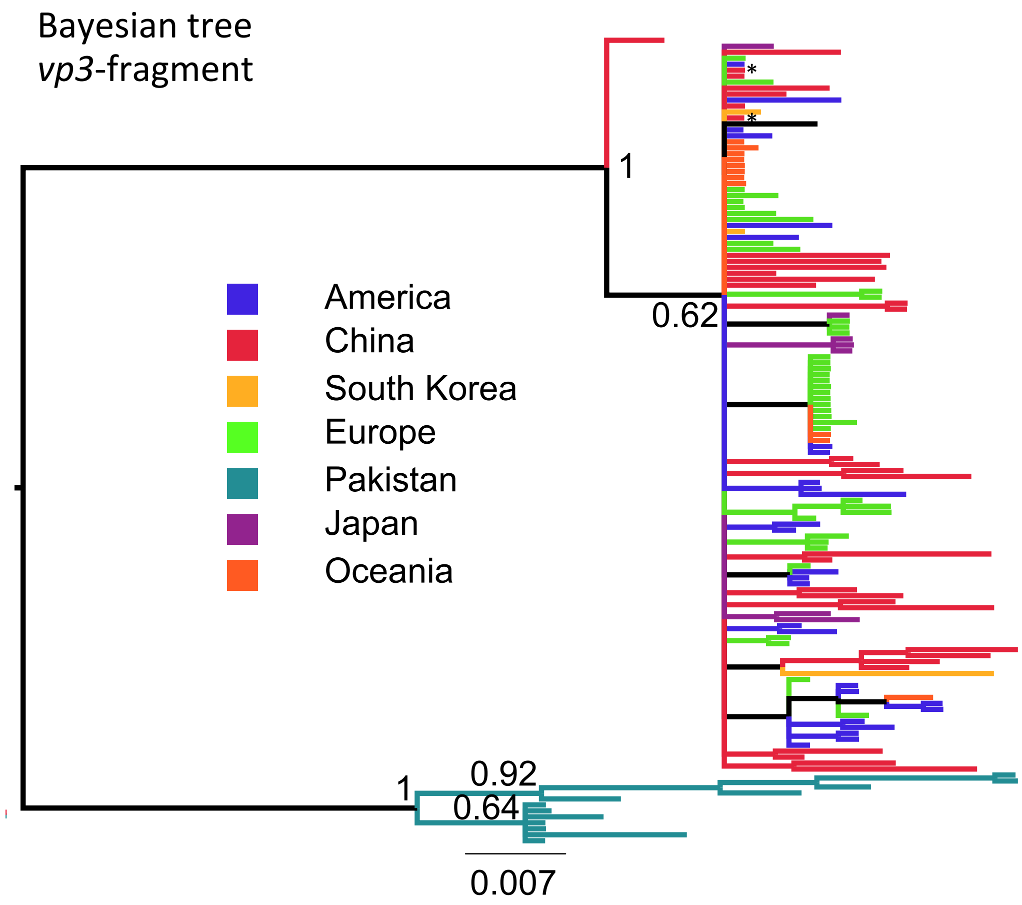

Fig. S3: MCC-tree of the lp-fragment. The colour gradient indicates the median of the α-rate parameter (evolutionary rate modeled via an exponential relaxed clock prior). Tip labels indicate the sequence name/Genbank accession number.

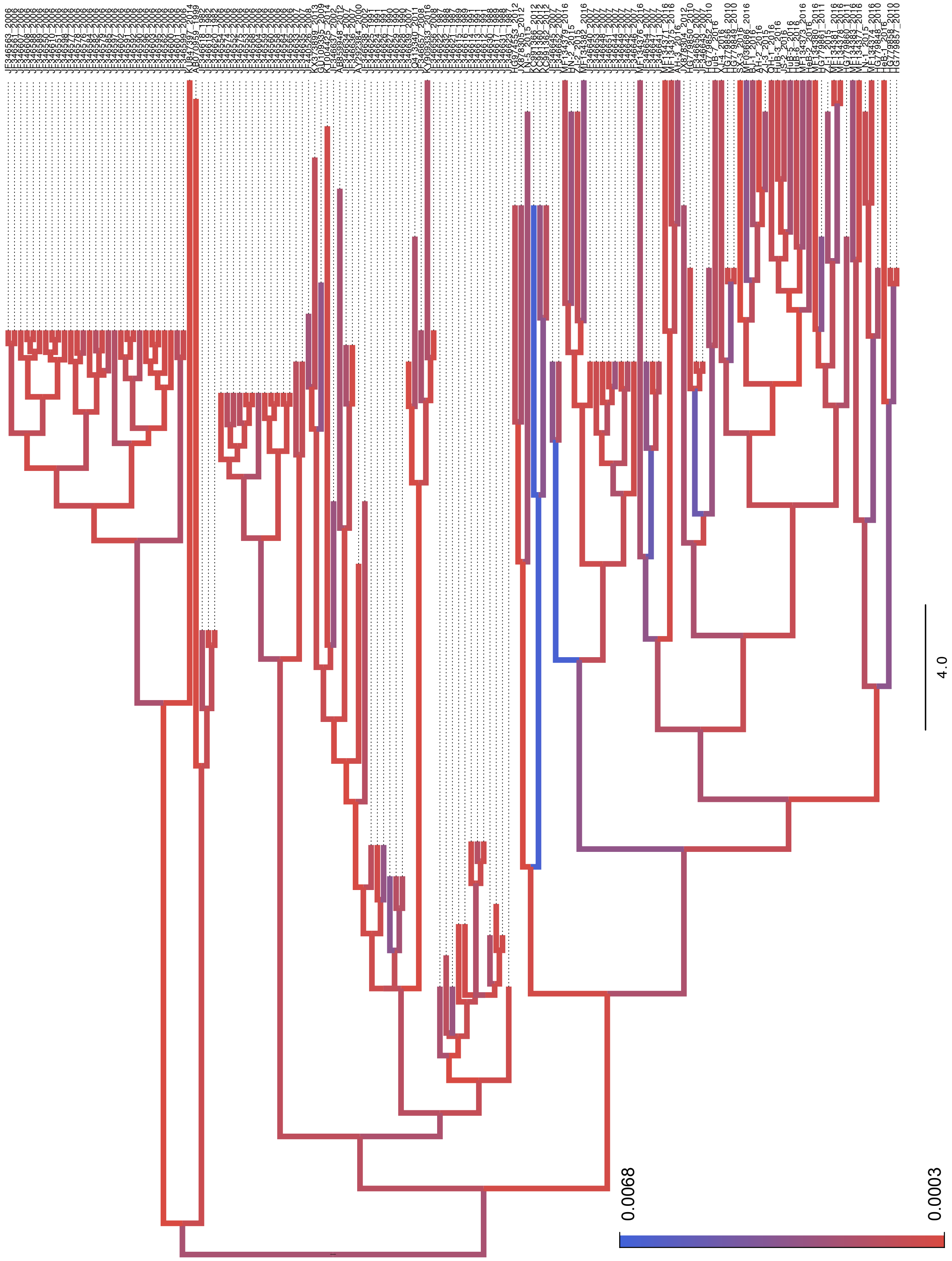

Fig. S4: MCC-tree of the rdrp-fragment. The colour gradient indicates the median of the α-rate parameter (evolutionary rate modeled via a lognormal relaxed clock prior). Tip labels indicate the sequence name/Genbank accession number.

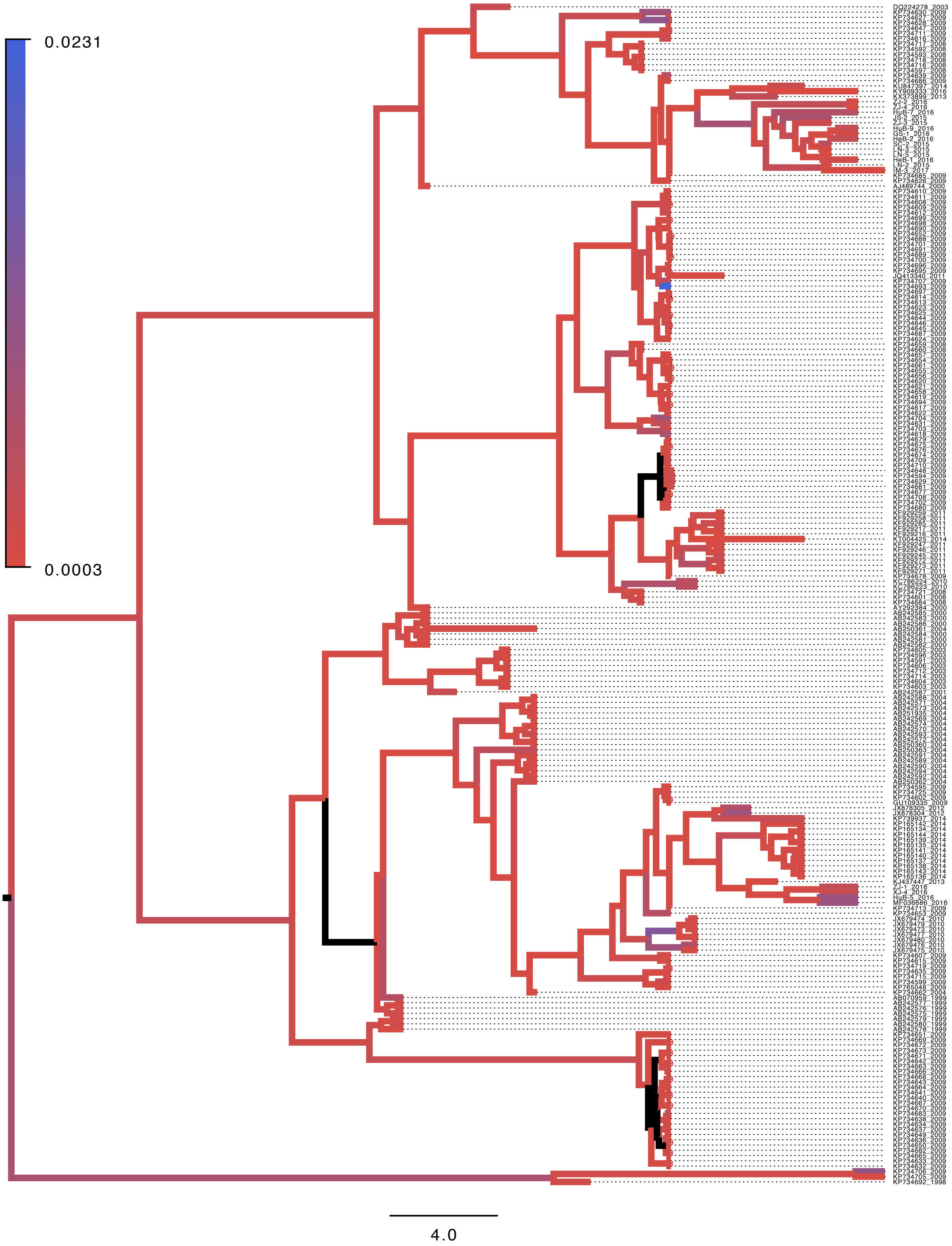

Fig. S5: Enlarged view of Fig. 2a – Bayesian tree *lp*-fragment

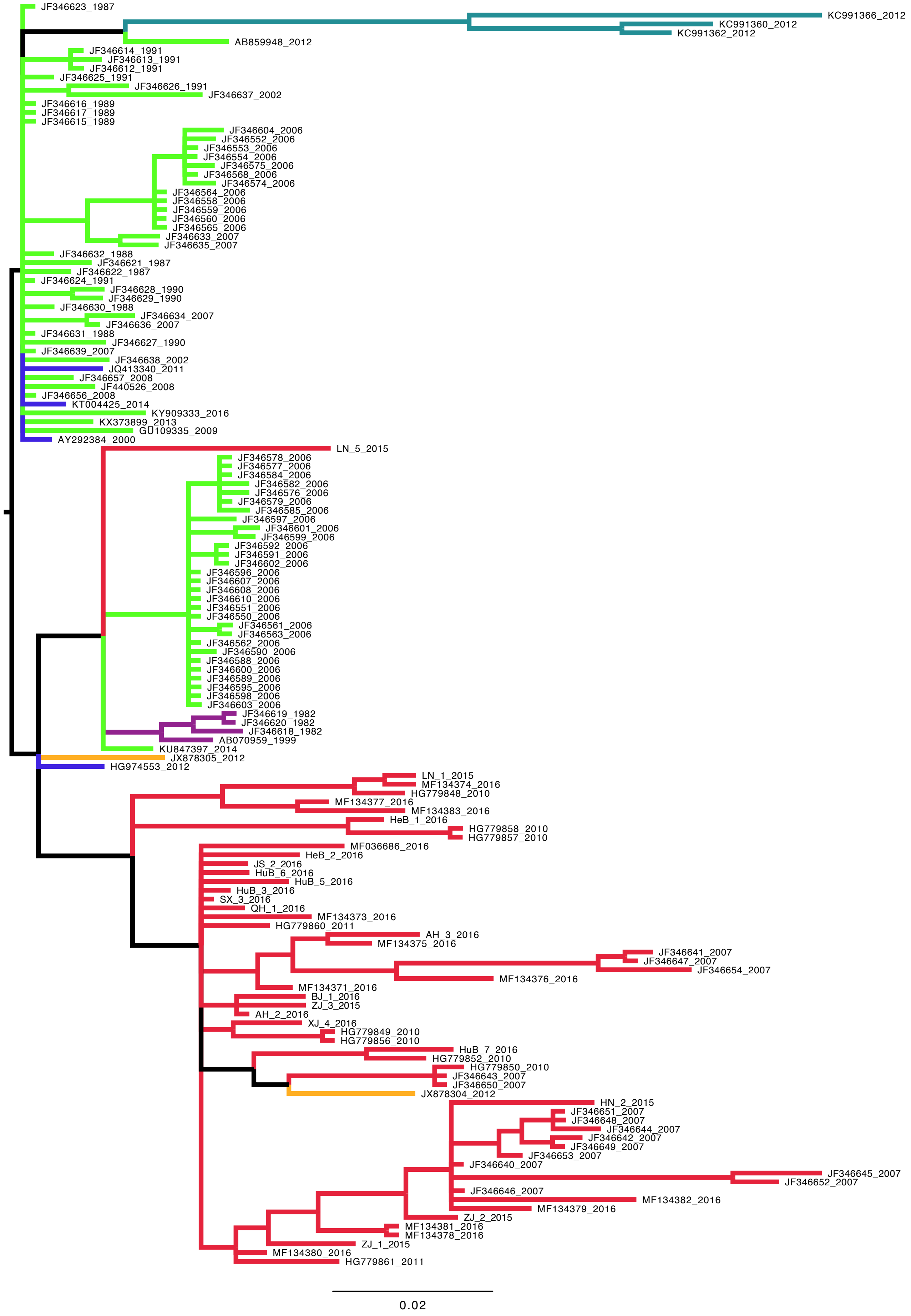

Fig. S6: Enlarged view of Fig. 2a – Bayesian tree *rdrp*-fragment

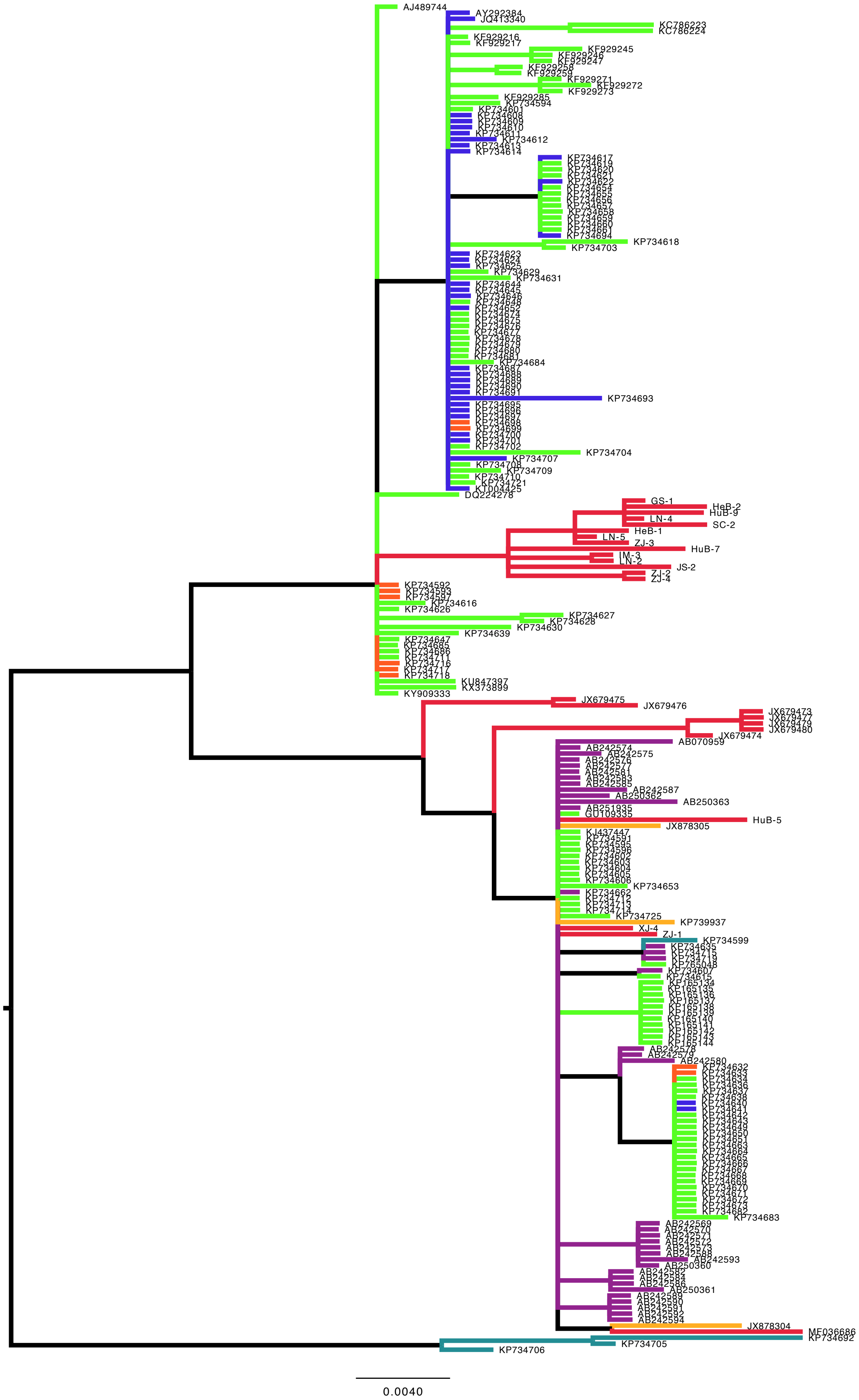

Fig. S7: Enlarged view of Fig. 2b – MCC tree *lp*-fragment

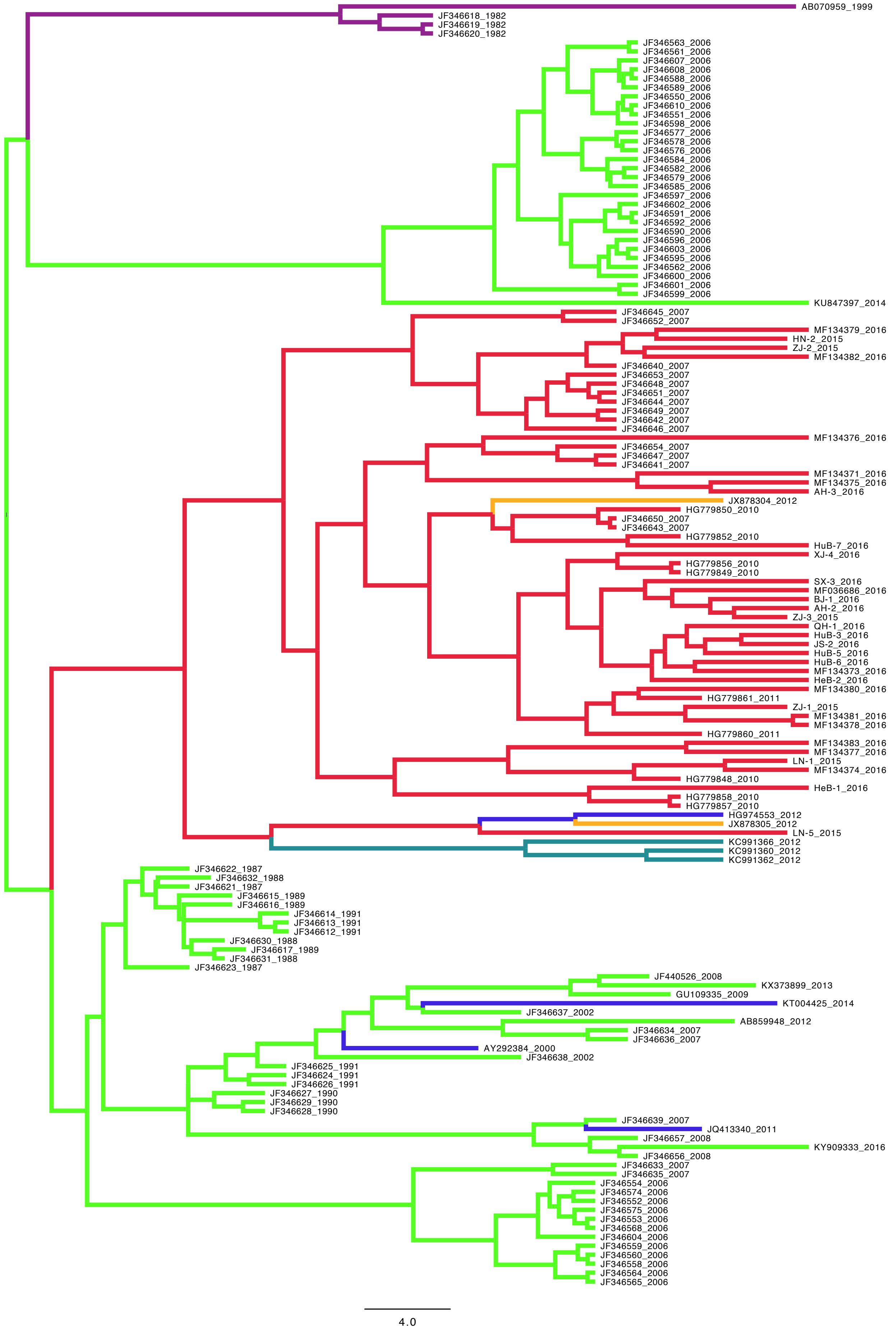

Fig. S8: Enlarged view of Fig. 2b – MCC tree *rdrp*-fragment

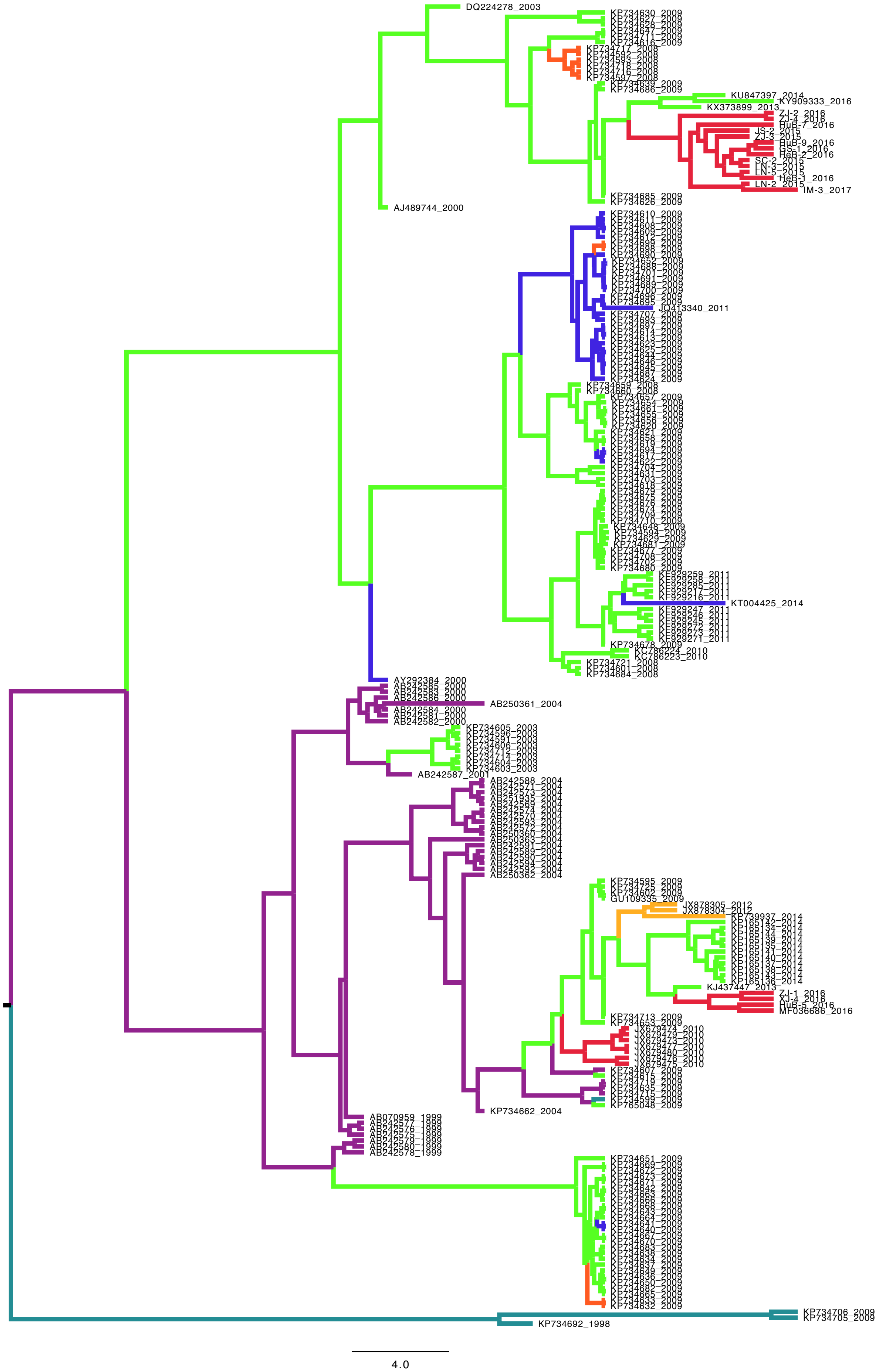

References

1 Yañez, O. *et al.* Potential for virus transfer between the honey bees *Apis mellifera* and *A. cerana*. *J. Api. Res.* **54,** 179-191; 10.1080/00218839.2015.1128145 (2015).

2 Ai, H. X., Yan, X. & Han, R. C. Occurrence and prevalence of seven bee viruses in *Apis mellifera* and *Apis cerana* apiaries in China. *J. Invertebr. Pathol.* **109,** 160-164; 10.1016/j.jip.2011.10.006 (2012).

3 Ding, G. *et al.* Prevalence of honeybee viruses in different regions of China and Argentina. *Rev. Sci. Tech. Off. Int. Epi.* **35,** 825-833; 10.20506/rst.35.3.2572 (2016).

4 Forsgren, E. *et al.* Preliminary observations on possible pathogen spill-over from *Apis mellifera to Apis cerana*. *Apidologie.* **46,** 265-275; 10.1007/s13592-014-0320-3 (2015).

5 Li, J. *et al.* The prevalence of parasites and pathogens in Asian honeybees *Apis cerana* in China. *PLoS ONE.* 7; 10.1371/journal.pone.0047955 (2012).

6 Kojima, Y. *et al.* Infestation of Japanese native honey bees by tracheal mite and virus from non-native European honey bees in Japan. *Microb. Ecol.* **62,** 895-906; 10.1007/s00248-011-9947-z (2011).

7 Choe, S. E. *et al.* Prevalence and distribution of six bee viruses in Korean *Apis cerana* populations. *J. Invertebr. Pathol.* **109,** 330-333; 10.1016/j.jip.2012.01.003 (2012).

8 Thu, H. T. *et al.* Prevalence of bee viruses among *Apis cerana* populations in Vietnam. *J. Api. Res.* **55,** 379-385; 10.1080/00218839.2016.1251193 (2016).

9 Wilfert, L. *et al.* Deformed wing virus is a recent global epidemic in honeybees driven by *Varroa* mites. *Science.* **351,** 594-597; 10.1126/science.aac9976 (2016).

10 Genersch, E. Development of a rapid and sensitive RT-PCR method for the detection of deformed wing virus, a pathogen of the honeybee (*Apis mellifera*). *Vet. J.* **169,** 121-123; 10.1016/j.tvjil.2004.01.004 (2005).

11 Yue, C. & Genersch, E. RT-PCR analysis of Deformed wing virus in honeybees (*Apis mellifera*) and mites (*Varroa destructor*). *J. Gen. Virol.* **86,** 3419-3424; 10.1099/vir.0.81401-0 (2005).
